# Diseases of the Bladder and Urethra in Women

**Published:** 1879-02

**Authors:** 


*Diseases of the Bladder and Urethra in Women.* By ALEXANDER J. C. SKENE, M. D., Professor of the Diseases of Women in the Long Island College Hospital, Fellow of the Gynecological Society of Boston, Member of the Medical Society of the County of Kings, and of the Obstetrical Society of New York. New York: William Wood & Co., 27 Great Jones street, 1878.

This work comprises eight lectures originally designed for the college class room, and intended to embody only those facts which the student and general practitioner require to know in the every-day demands of active practice. These lectures have been revised and enlarged to constitute a complete text-book upon the Functional Anomalies of the Bladder and Urethra.

Wood cut illustrations simplify the text and add to the value of the work, which we judge will be received and appreciated by the profession as a text-book guide in general practice.

